# Prognostic impact of treatment‐related and geriatric factors in older patients with classic Hodgkin lymphoma: A real‐life cohort study

**DOI:** 10.1111/bjh.70390

**Published:** 2026-02-20

**Authors:** Silvio Ligia, Giovanni Manfredi Assanto, Tania Soriano, Luca Vincenzo Cappelli, Mauro Passucci, Giorgia Annechini, Gianna Maria D'Elia, Alessandro Pulsoni, Maurizio Martelli, Ilaria Del Giudice

**Affiliations:** ^1^ Hematology, Department of Translational and Precision Medicine Sapienza University of Rome Rome Italy; ^2^ Hematology AOU Policlinico Umberto I Rome Italy; ^3^ Hematology Unit S. Maria Goretti Hospital Latina Italy; ^4^ Present address: Division of Hematology, Department of Medicine McMaster University Hamilton Ontario Canada

**Keywords:** ACA index, Frailty Score, geriatric assessment, Hodgkin lymphoma, older patients

## Abstract

Older patients with classic Hodgkin lymphoma experience poorer outcomes than younger individuals, due to comorbidities and functional limitations affecting treatment tolerance. In this retrospective single‐centre study of 140 patients, we evaluated treatment patterns, outcomes and the prognostic value of two geriatric scores: the Age, Comorbidities and Albumin (ACA) Index and the Frailty Score. Most patients (87.9%) received standard ABVD (doxorubicin, bleomycin, vinblastine and dacarbazine)/AVD (doxorubicin, vinblastine and dacarbazine) regimens. At median follow‐up (65 months), 5‐year progression‐free survival (PFS) and overall survival (OS) were 65% and 78.5%, respectively; treatment‐related mortality was 7.8%. First‐line therapy (AVD‐based versus other) significantly influenced PFS (61.8% vs. 23.5%, *p* = 0.002) and OS (69.1% vs. 35.3%, *p* = 0.001). ACA Index stratified patients into four risk categories with progressively lower 5‐year OS (92%–64.3%, *p* = 0.003). According to Frailty Score, 37.9% were fit, 54.3% unfit and 7.8% frail, with corresponding 5‐year OS of 79.2%, 59.2% and 36.4% (*p* < 0.0001). Multivariate analysis identified age ≥80 years, extra‐nodal involvement and advanced stage according to German Hodgkin Study Group (GHSG) classification as independent predictors of PFS; age ≥80 years, advanced stage (GHSG) and Frailty Score independently predicted OS. Our findings confirm that standard therapies achieve favourable outcomes in older patients, but chronological age alone is insufficient for treatment decisions. The Frailty Score offers an accessible prognostic tool to guide therapy, supporting the integration of geriatric assessment into treatment planning for this underrepresented population.

## INTRODUCTION

Patients with classic Hodgkin lymphoma (cHL) are typically considered ‘older’ when diagnosed at ≥60 years. Outcomes differ significantly by age, with 5‐year overall survival (OS) exceeding 85% in younger patients but dropping to 50%–75% in older ones.^1^ This disparity reflects an unmet medical need, since distinctive biological features, comorbidities, and impaired functional or nutritional status limit older patients' eligibility and tolerance to standard treatments.^2^, ^3^

In elderly patients with diffuse large B‐cell lymphoma (DLBCL), the Fondazione Italiana Linfomi (FIL) proposed a simplified geriatric assessment (sGA)—based on age, activities of daily living (ADL) and the Cumulative Illness Rating Scale‐Geriatric (CIRS‐G)—to classify patients ≥65 years of age as fit, unfit or frail and this correlated with OS.^4^, ^5^ The sGA, combined with the International Prognostic Index and haemoglobin levels, form the new Elderly Prognostic Index, validated for mortality prediction in DLBCL.^5^ Contrariwise, no such validated tool exists for older cHL patients. The International Prognostic Score, although widely used for cHL patients, is inadequate for prognosis prediction in older patients.^6^

The ACA index—incorporating age, comorbidities and serum albumin—has been proposed in older DLBCL patients and categorized them into four risk groups (excellent, good, moderate and poor), with 3‐year OS rates of 86%, 72%, 51% and 0% respectively. This index also predicted treatment tolerability and adherence to R‐CHOP (rituximab, cyclophosphamide, doxorubicin, vincristine, prednisone).^7^ Its predictive value for OS was confirmed in an independent cohort of elderly DLBCL.^8^ The ACA index can be retrospectively applicable, but it has not yet been tested in patients with other types of lymphomas, including cHL. More recently, the Nordic Lymphoma Group (NLG) developed a simplified Frailty Score based on age, comorbidities and Eastern Cooperative Oncology Group (ECOG) Performance Status (PS), specifically for older cHL patients.^9^ By categorizing patients as fit, unfit or frail, this tool not only predicted markedly different 5‐year OS (86%, 52% and 22%, respectively) and progression‐free survival (PFS) (74%, 49% and 11%, respectively) but also provided a practical framework to guide treatment intensity and individualized therapeutic decisions in this vulnerable population.^9^

This study aims to retrospectively evaluate treatment strategies and outcomes in older cHL patients treated at our Institution over two decades and to assess the prognostic value of the ACA index and the Frailty Score in predicting PFS and OS.

## PATIENTS AND METHODS

This retrospective, single‐centre study initially included 154 patients aged ≥60 years with newly diagnosed Hodgkin lymphoma (HL), consecutively treated at our Institute between 2000 and November 2022: 140 (90.9%) had cHL and 14 (9.1%) nodular lymphocyte‐predominant HL (NLPHL). The latter 14 NLPHL patients were excluded from the final data analysis. Data were extracted from medical charts, including clinical characteristics (stage, ECOG‐PS, B symptoms, bulky/extra‐nodal disease), laboratory values at diagnosis, first‐line treatment, response, adverse events, toxicities (haematological, cardiac, neurological, infectious and bleomycin‐related lung toxicity [BLT]) and follow‐up. Comorbidities were retrospectively evaluated individually and via the CIRS‐G scale.^4^ According to the ACA Index, we assigned one point each for age ≥75 years, hypoalbuminaemia (<3.7 g/dL) and high burden of comorbidities (Charlson Comorbidity Index [CCI] ≥3).^7^, ^8^ Patients were stratified into four risk groups: excellent (0 points), good (1), moderate (2) and poor (3). According to the NLG Frailty Score, based on age ≥70, ECOG‐PS ≥2 and CIRS‐G ≥8, we classified patients as fit (0), unfit (1–2) or frail (3).^9^ Lymphoma diagnosis and its impact on organ function have not been considered for CIRS‐G and CCI calculation. Treatment‐related mortality (TRM) was defined as death of any cause during or within 3 months after first‐line treatment, not clearly due to cHL. PFS was defined from the time of treatment initiation to progression, relapse or death for any cause (whichever comes first) and OS considered as time from diagnosis to death for any cause. Details about the statistical analysis are provided in Supporting Information.

## RESULTS

### Baseline characteristics of patients

From a total of 154 older HL patients initially collected, 140 (90.9%) had cHL. The median age at diagnosis was 69 years (range 60–89 years); 83 (59.3%) patients were males and 57 (40.7%) females. At diagnosis, 63 patients (45%) had stage I–II disease and 77 (55%) stage III–IV; bulky disease (≥7 cm) was present in 17 cases (12.1%) and B symptoms in 65 (46.4%). According to the German Hodgkin Study Group (GHSG) classification,^10^ 64 patients (45.7%) had early‐stage disease—33 (23.6%) favourable, 31 (22.1%) unfavourable—and 76 (54.3%) had advanced‐stage disease. Most patients (68.6%) had ECOG PS 0–1. Table 1 shows the clinical and laboratory features by age groups. Patients aged ≥80 years more frequently presented B symptoms (*p* = 0.05), ECOG PS 2–4 (*p* < 0.001), CIRS‐G ≥8 (*p* = 0.009), albumin level <3.5 g/dL (*p* = 0.027) and mixed cellularity or lymphocyte‐rich subtypes (*p* = 0.05).

**TABLE 1:** Patients' characteristics at diagnosis.

Characteristics		Age category								*p*‐value
		60–69 years (*N* = 71)		70–79 years (*N* = 50)		≥80 years (*N* = 19)		Total (*N* = 140)		
		*N*	%	*N*	%	*N*	%	*N*	%	
Sex	Male	43	60.6%	29	58.0%	11	57.9%	83	59.3%	0.952
	Female	28	39.4%	21	42.0%	8	42.1%	57	40.7%	
Histological subtype	NS	39	54.9%	20	40.0%	6	31.6%	65	46.4%	**0.05**
	MC	25	35.2%	18	36.0%	10	52.6%	53	37.8%	
	LR	3	4.2%	8	16.0%	2	10.5%	13	9.3%	
	LD	0	0.0%	0	0.0%	0	0.0%	0	0.0%	
	Unspecified	4	5.6%	4	8.0%	1	5.3%	9	6.4%	
Stage	I–II	30	42.3%	27	54.0%	6	31.6%	63	45.0%	0.19
	III–IV	41	57.7%	23	46.0%	13	68.4%	77	55.0%	
GHSG	Favourable	16	22.5%	13	26.0%	4	21.1%	33	23.6%	0.47
	Unfavourable	15	21.1%	14	28.0%	2	10.5%	31	22.1%	
	Advanced	40	56.3%	23	46.0%	13	68.4%	76	54.3%	
Presence of B symptoms		34	47.9%	18	36.0%	13	68.4%	65	46.4%	**0.05**
Bulky disease (≥7 cm)		12	16.9%	3	6.0%	2	10.5%	17	12.1%	0.19
Hepatic involvement		3	4.2%	2	4.0%	2	10.5%	7	5.0%	0.49
Spleen involvement		18	25.4%	12	24.0%	7	36.8%	37	26.4%	0.53
Bone marrow involvement		14	19.7%	6	12.0%	2	10.5%	22	15.7%	0.41
Extra‐nodal disease		6	8.5%	2	4.0%	1	5.3%	9	6.4%	0.60
ECOG‐PS	0–1	56	78.9%	37	74.0%	3	15.8%	96	68.6%	**<0.001**
	2–4	15	21.1%	13	26.0%	16	84.2%	44	31.4%	
Albumin	≤3.5 g/dL	23	35.4%	6	13.0%	6	33.3%	35	27.1%	**0.027**
	>3.5 g/dL	42	64.6%	40	87.0%	12	66.7%	94	72.9%	
CIRS‐G score	<8	65	91.5%	41	82.0%	12	63.2%	118	84.3%	**0.009**
	≥8	6	8.5%	9	18.0%	7	36.8%	22	15.7%	
Tobacco smoke		23	32.4%	10	20.0%	4	21.1%	37	26.4%	0.26
Leucocytosis	>11 × 10^9^/L	12	16.9%	10	20.4%	4	21.1%	26	18.7%	0.85
Anaemia	<11 g/dL	13	18.3%	8	16.3%	3	15.8%	24	17.3%	0.94
ESR	>50 mm/h	21	29.6%	16	32.0%	8	42.1%	45	32.1%	0.58

*Note*: A *p*‐value ≤0.05 was considered statistically significant are in bold.

Abbreviations: CIRS‐G, cumulative illness rating scale in geriatrics; ECOG‐PS, Eastern Cooperative Oncology Group performance status; ESR, erythrocyte sedimentation rate; GHSG, German Hodgkin Study Group; LD, lymphocyte‐depleted; LR, lymphocyte‐rich; MC, mixed cellularity; NS, nodular sclerosis.

Comorbidities were present in 124 (88.6%) patients at diagnosis; the remaining 16 patients without comorbidities were all aged 60–69 years. Full comorbidity data are provided in Table S1.

The median CIRS‐G was 4 (range 0–14), increasing significantly with age (median 3 in 60–69 years, 5 in 70–79, and 6 in ≥80; *p* = 0.0028). Overall, in 29 patients (20.7%), CIRS‐G was >6; in 22 (15.7%), it was ≥8; and in 7 (5%), it was ≥10.

### First‐line treatment

All patients underwent first‐line treatment. Of these, 123 (87.9%) received ABVD (doxorubicin, bleomycin, vinblastine and dacarbazine) or AVD (doxorubicin, vinblastine and dacarbazine)‐based regimens, 17 (12.1%) received alternative regimens (procarbazine, vinblastine, cyclophosphamide, prednisone [PROVECIP]; vinblastine, cyclophosphamide, procarbazine, etoposide, mitoxantrone, bleomycin [VEPEMB]; radiotherapy [RT] only [3, 2.1%]). Among the 123 patients treated with ABVD/AVD‐based regimens, four received AVD (3.3%) due to pre‐existing severe pneumopathies, 16 (13%) received two cycles of ABVD followed by four AVD after negative interim positron‐emission tomography/computed tomography (PET/CT) scan, three received brentuximab vedotin (BV) + AVD (2.4%) and 100 (81.3%) received full ABVD. Among the latter, 14 patients received only two ABVD cycles (12 early‐stage disease and 2 TRM), 35 received three to four cycles (30 early‐stage disease and 5 early treatment discontinuation) and five patients discontinued bleomycin within the first two to three cycles due to early‐onset BLT (2 patients) or clinical judgement. Bleomycin was not omitted in 46 advanced‐stage patients, who received more than two cycles of the full ABVD regimen.

Overall, 69 patients (49.3%) received combined chemotherapy and RT, 79% of them with early‐stage and 21% with advanced‐stage disease.

Treatment cycles' delays occurred in 60 patients (43.5%), 16 of whom later relapsed or had refractory disease. Dose reductions were needed in 20 patients (14.6%), and early treatment discontinuation occurred in 22 (16.2%), both events being more frequent in patients ≥80 years (Table S2).

### Treatment‐related complications and acute toxicity

Infectious complications were the most frequent adverse events during first‐line therapy, affecting 74 patients (54.0%), with significantly higher rates in older age groups (*p* = 0.05; Table 2). Pneumonia occurred in 28 patients (20.4%). Among patients treated with bleomycin‐containing regimens, 12/124 (9.7%) developed BLT, with no significant difference across age groups (*p* = 0.95); two had early‐onset BLT (within the first 2–3 cycles). Grade 3–4 neutropenia occurred in 86 patients (62.8%), significantly more often in older patients (*p* = 0.04). Overall, (granulocyte colony‐stimulating factor) G‐CSF prophylaxis was administered in 102 patients (72.8%). Erythropoiesis‐stimulating agents were used in 19 patients (13.5%), and 12 (9.8%) developed grade 3–4 anaemia requiring red blood cell transfusions. Acute cardiovascular toxicity occurred in eight patients (5.8%), including one fatal event. Grade 3–4 peripheral neurotoxicity was observed in five patients (3.7%). TRM rate was 7.8% (*n* = 11), including four patients early lost to follow‐up and presumed deceased (Table S3).

**TABLE 2:** Treatment‐related toxicities according to age groups.

Adverse event	Age categories								*p*‐value
	60–69 years (*N* = 71)		70–79 years (*N* = 50)		≥80 years (*N* = 19)		Total (*N* = 140)		
	*N*	%	*N*	%	*N*	%	*N*	%	
Hospitalizations	10	15.9%	10	22.7%	5	31.3%	25	20.3%	0.35
Infections	34	48.6%	26	53.1%	14	77.8%	74	54.0%	**0.05**
Pneumonia	12	17.1%	9	18.4%	7	38.9%	28	20.4%	0.11
Bleomycin‐related lung toxicity (BLT)	7	10.1%	4	8.7%	1	8.3%	12	9.4%	0.95
Bleeding event	2	2.9%	2	4.1%	1	5.6%	5	3.6%	0.84
Blood transfusion required	4	6.5%	4	9.1%	4	25.0%	12	9.8%	0.08
Haematological toxicity G3–4	37	52.9%	36	73.5%	13	72.2%	86	62.8%	**0.04**
Neurological Toxicity G3–4	2	2.9%	2	4.2%	1	5.6%	5	3.7%	0.84
Cardiac toxicity G3–5	1	1.4%	6	12.2%	1	5.6%	8	5.8%	**0.04**

*Note*: A *p*‐value ≤0.05 was considered statistically significant are in bold.

### Response to first‐line treatment

At the end of first‐line treatment (EOT), 107 patients (82.4%) achieved a complete response (CR), 18 (13.8%) a partial response, 1 (0.8%) had stable disease and 4 (3.1%) had progressive disease. EOT response was assessed by PET/CT scan in 90/130 cases (69.2%). Response was not assessable in 10 patients due to six early deaths (TRM) and four early lost to follow‐up.

Interim PET/CT scan (PET2) was performed in 94 patients (67.1%), and 85 (90.4%) were negative. Deauville Score (DS) was assessed in 65 cases: 61 (93.8%) had DS 1–3, and 4 (6.2%) had DS 4–5. Among PET2‐positive patients (all treated with ABVD), no treatment intensification was implemented, and bleomycin was not omitted. Patients treated with AVD‐based regimens had a significantly higher CR rate (87.1%), compared to those receiving other regimens (46.7%) (*p* = 0.001).

### PFS and OS—Univariate analysis

At a median follow‐up of 65 months (range 2–220 months), PFS was 57.1% (patients at risk of events: 80/140) and OS 65% (91/140). PFS at 2 and 5 years was 75.7% (106/140) and 65% (91/140) respectively. OS at 2 and 5 years was 87.8% (123/140) and 78.5% (110/140) respectively.

**FIGURE 1:**
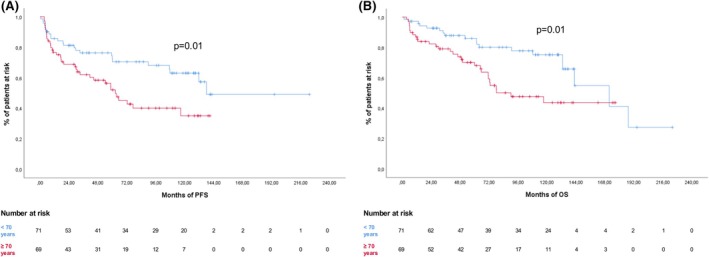
Progression‐free survival (PFS) (A) and overall survival (OS) (B) according to age (<70 years, blue line; ≥70 years, red line). [Colour figure can be viewed at wileyonlinelibrary.com]

Univariate analysis showed a significantly worse PFS for: age ≥70 years (47.8%) versus <70 (66.2%) (*p* = 0.01; Figure 1A), ECOG‐PS 2–4 (40.9%) versus 0–1 (64.6%) (*p* = 0.002), B symptoms present (46.2%) versus absent (66.7%) (*p* = 0.004), stage III–IV (48.1%) versus I–II (68.3%) (*p* = 0.003), splenomegaly (45.9%) versus no splenomegaly (61.2%) (*p* = 0.008), extra‐nodal disease (35.6%) versus nodal‐only (78.8%) (*p* = 0.001) and GHSG risk groups (advanced 48.7% vs. early‐unfavourable 54.8% vs. early‐favourable 78.8%) (*p* = 0.004).

Regarding OS, beside age (*p* = 0.011; Figure 1B), ECOG‐PS (*p* = 0.001), B symptoms (*p* = 0.05), stage (*p* = 0.001), GHSG risk groups (<0.001), also CIRS‐G ≥8 (OS 45.5%) had a significant impact compared to CIRS‐G <8 (OS 68.6%) (*p* < 0.0001).

**FIGURE 2:**
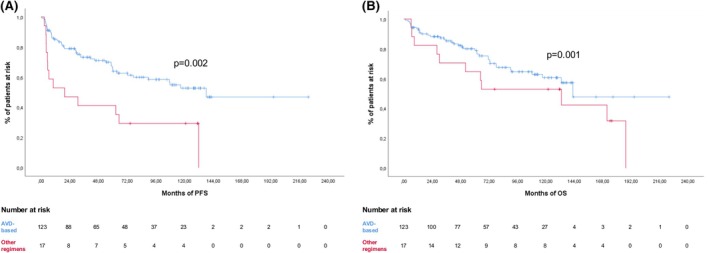
Progression‐free survival (PFS) (A) and overall survival (OS) (B) according to therapy (AVD [doxorubicin, vinblastine and dacarbazine]‐based, blue line; other regimens, red line). [Colour figure can be viewed at wileyonlinelibrary.com]

The type of first‐line therapy, comparing AVD‐based regimens (*n* = 123) versus other therapies (*n* = 17), significantly influenced both PFS (61.8% vs. 23.5%, *p* = 0.002) and OS (69.1% vs. 35.3%, *p* = 0.001) (Figure 2A,B).

Among treatment‐related complications, infections—particularly pneumonias—showed a marked negative impact on prognosis. Patients who developed pneumonia had significantly lower PFS (28.6% vs. 65.1%) and OS (38.9% vs. 76.9%) compared to those without this complication (*p* < 0.001 for both). Additional factors associated with inferior PFS included the absence of CR at EOT (*p* < 0.0001), delays in therapy cycles (*p* = 0.014) and early treatment discontinuation (*p* < 0.0001). Similarly, OS was adversely affected by failure to achieve CR at EOT (*p* = 0.001), occurrence of at least one hospitalization during treatment (*p* = 0.04) and early discontinuation (*p* < 0.0001). Other toxicities, including BLT, cardiovascular complications, grade 3–4 haematological toxicity or neurotoxicity, did not show a statistically significant impact on survival outcomes.

### Patient‐ and lymphoma‐related independent predictors of PFS and OS

In the multivariate analysis (MVA) including both patient‐related and disease‐specific variables (before treatment), age ≥80 years (compared to 60–69 years), the presence of extra‐nodal disease and GHSG risk classification—which encompasses stage and systemic symptoms—independently predicted shorter PFS. Specifically, age ≥80 was associated with nearly doubled risk of progression (hazard ratio [HR] 1.94, 95% confidence interval [CI] 1.39–2.71, *p* < 0.0001), extra‐nodal involvement had a HR of 2.5 (95% CI 1.1–5.6 *p* = 0.027) and advanced stage according to GHSG classification was also significant (HR 1.66, 95% CI 1.16–2.34, *p* = 0.005), as reported in Table S4.

For OS, age ≥80 years remained a strong independent predictor of mortality (HR 3.3, 95% CI 1.54–7.1, *p* = 0.002), as did advanced disease (GHSG; HR 1.65, 95% CI 1.1–2.5, *p* = 0.017). Importantly, the Frailty Score retained independent prognostic significance (HR 2.21, 95% CI 1.17–4.17, *p* = 0.014), highlighting its additive prognostic value beyond chronological age (Table S5).

### Treatment‐related predictors of PFS and OS

Among treatment‐related factors, achieving a CR at EOT emerged as the strongest independent predictor of both PFS (HR 3.36, 95% CI 2.30–4.90, *p* < 0.001) and OS (HR 2.9, 95% CI 1.86–4.48, *p* < 0.001), underscoring the prognostic relevance of response depth even in older patients. Failure to achieve CR was associated with the presence of B symptoms (HR 4.09, *p* = 0.027), extra‐nodal disease (HR 5.05, *p* = 0.007) and use of non‐AVD‐based first‐line regimens (HR 9.03, *p* < 0.001). Chemotherapy delays had a modest but significant effect on PFS (HR 1.88, 95% CI 1.01–3.50, *p* = 0.048), though they did not significantly influence OS.

The occurrence of pneumonia during first‐line treatment was significantly associated with inferior survival (HR 2.37, 95% CI 1.24–4.53, *p* = 0.009), likely reflecting both patient frailty and treatment toxicity.

Relapse had a strong impact on prognosis: 2‐year OS was 97% in patients without relapse, 85% in those relapsing after 6 months and only 39% in early relapses (<6 months) (*p* < 0.0001).

Secondary malignancies were diagnosed in 19 patients (13.5%) during follow‐up and were associated with significantly reduced survival, with a 2‐year OS 31.6% compared to 70.2% in patients without secondary cancers (*p* = 0.012).

### Prognostic scores evaluation

**FIGURE 3:**
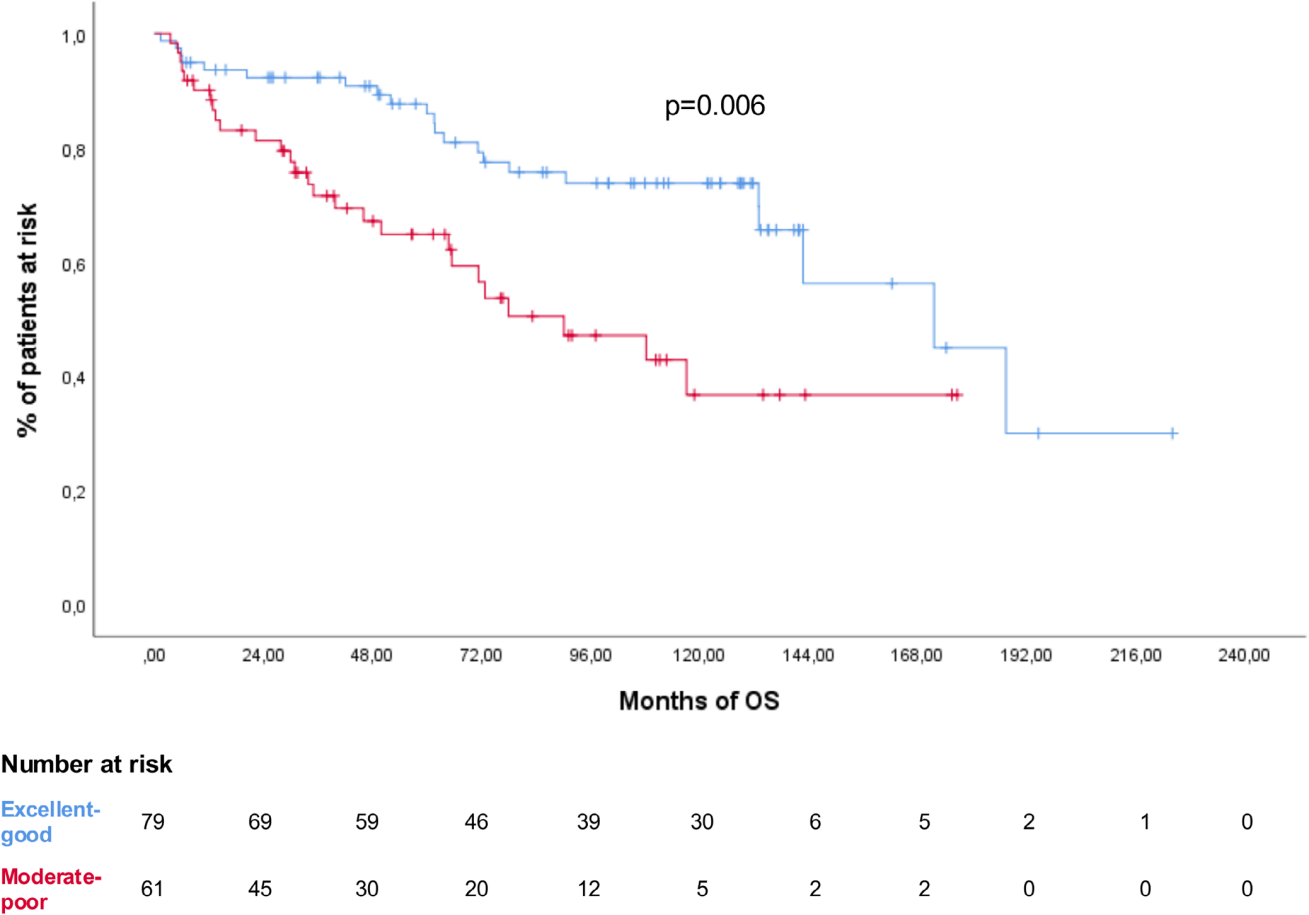
Overall survival (OS) in the entire cohort according to the ACA score at diagnosis (excellent‐good, blue line; moderate‐poor, red line). [Colour figure can be viewed at wileyonlinelibrary.com]

The ACA score was assessable in all patients: 25 (17.9%) had an excellent score, 54 (38.6%) good, 47 (33.6%) moderate and 14 (10%) poor. Five‐year OS declined progressively across risk categories (92%, 85.2%, 70.2% and 64.3%, respectively; *p* = 0.003). A moderate‐poor ACA score was significantly associated with early treatment discontinuation (*p* = 0.017), higher incidence of infections (*p* = 0.05) and inferior 5‐year OS compared to excellent‐good scores (55.7% vs. 72.2%; *p* = 0.006) (Figure 3). This prognostic effect was confirmed in the subgroup of patients treated with AVD‐based regimens (OS 63.6% for moderate‐poor scores, vs. 75.6% for excellent‐good scores; *p* = 0.02).

**FIGURE 4:**
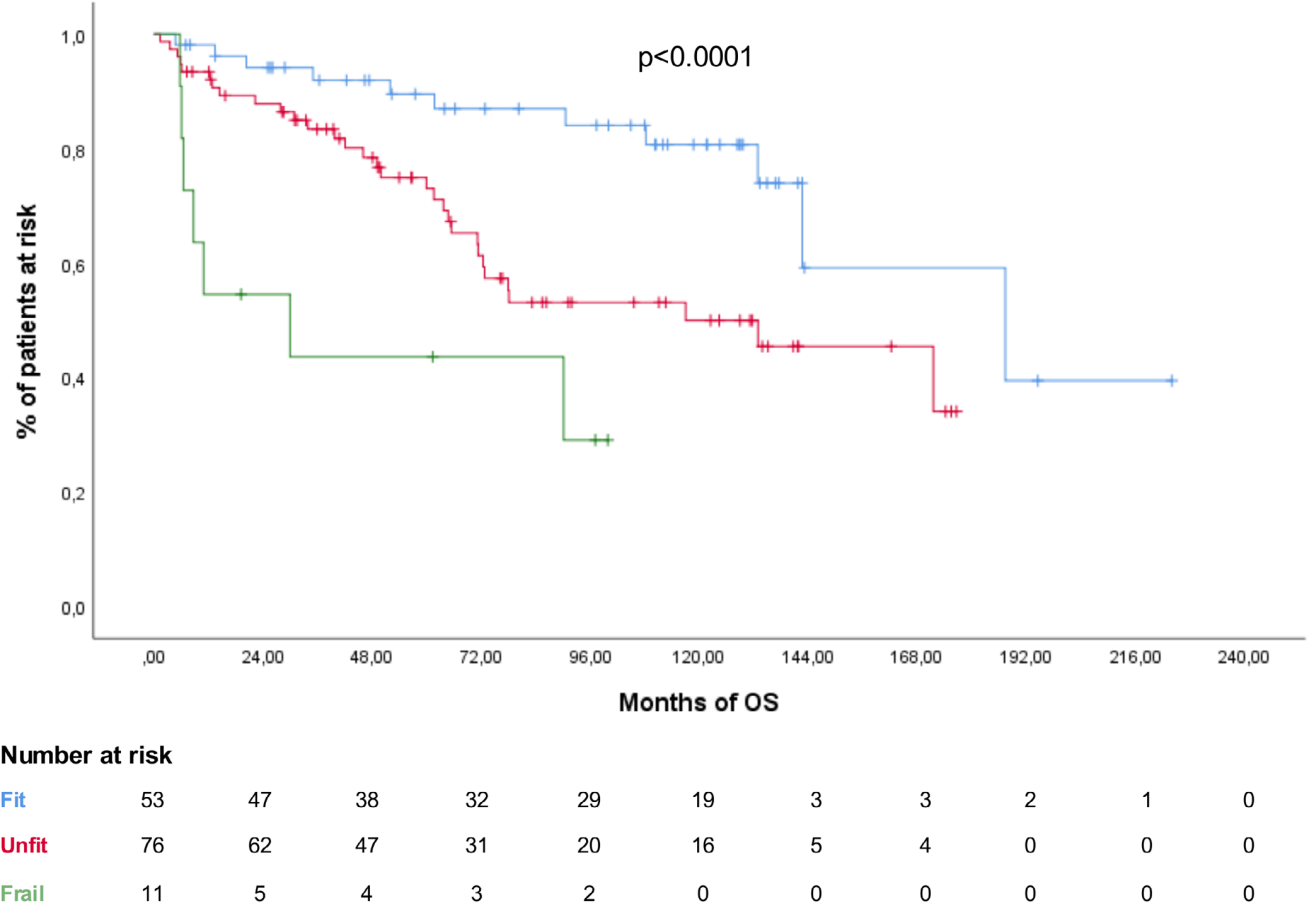
Overall survival (OS) in the entire cohort according to the Frailty score at diagnosis (fit, blue line; unfit, red line; frail, green line). [Colour figure can be viewed at wileyonlinelibrary.com]

According to the Frailty Score, 53 (37.9%) patients were classified as fit, 76 (54.3%) as unfit and 11 (7.8%) as frail. Five‐year OS significantly worsened with increasing frailty, with rates of 79.2%, 59.2% and 36.4% respectively (*p* < 0.0001) (Figure 4). The differences in OS rates of the Frailty score risk groups remained significant also in the AVD‐treated cohort (*p* = 0.002).

## DISCUSSION

Although cHL is typically considered a disease of the young, epidemiological data show that 15%–30% of patients are aged ≥60 years. In this group, outcomes remain suboptimal, with 5‐year OS rates between 50% and 75%, markedly lower than in younger patients.^1^, ^3^, ^11^ Their underrepresentation in randomized trials limits the availability of evidence‐based treatment guidelines. This retrospective study analysed 140 older cHL patients diagnosed and treated at our centre from 2000 onwards, assessing how age and other clinical factors impacted management and prognosis. The median age at diagnosis was 69 years; one‐third were aged 70–79 years and 13% between 80 and 89 years, similar to other real‐world series.^2^ Rates of B symptoms (46.4%) and advanced‐stage disease (55%) were high, while bulky (12.1%) and extra‐nodal involvement (6.4%) were relatively infrequent, consistent with previous reports.^3^, ^11^, ^12^ Histologically, nodular sclerosis cHL (46.4%) but also mixed cellularity cHL (37.8%) were common, confirming potential biological differences in older HL.^1^, ^13^

At diagnosis, 88.6% patients had ≥1 comorbidity, most frequently cardiovascular (62.1%), pulmonary (28.6%) and diabetes (22.1%). The median CIRS‐G score was 4 and increased with age. Typically, over 50% of older patients have at least one severe comorbidity.^14^ In a recently published multicentric retrospective study assessing the fitness of 244 elderly HL patients, Orellana‐Noia et al. reported a median CIRS‐G of 5 (range 0–22), similar to our cohort, and showed that scores ≥10—observed in 18% of patients—were associated with worse OS (*p* = 0.03).^15^ Similarly, Evens et al. found that 61% of elderly HL patients had at least one severe comorbidity, and 46% had a CIRS‐G score >6, predictive of poorer OS (*p* = 0.047).^2^ In our cohort, comorbidities were frequent but generally less severe: 29 patients (20.7%) had CIRS‐G >6, 22 (15.7%) ≥8 and only 7 (5%) ≥10. A CIRS‐G ≥8 was associated with worse OS (*p* = 0.0001), while thresholds of >6 (*p* = 0.20) or ≥10 (*p* = 0.15) were not predictive. Unlike the aforementioned studies, we could not assess ADL impairment due to the retrospective design of the study, and this represents an important limitation, as dependency in ADL has been shown to be a strong predictor of OS and PFS.^2^, ^15^ Indeed, Evens et al. proposed a prognostic model combining ADL impairment and age ≥70 years, which—based on the number of these risk factors (0, 1, or 2)—stratified patients by 5‐year OS (73%, 51%, 0%) and PFS (55%, 39%, 0%) (*p* < 0.0001).^11^

To overcome this limitation, we applied two scores that make possible the retrospective evaluation of comorbidities. First, the ACA index, proposed for DLBCL^7^, ^8^ and never tested in older cHL. In our cohort, the ACA Index stratified patients into four risk categories with progressively worsening 5‐year OS (92%–64.3%, *p* = 0.003). Furthermore, we tested the Frailty Score, recently developed and validated by the NLG for older cHL treated with anthracycline‐based regimens.^9^ The NLG cohort included 279 patients diagnosed between 2000 and 2015, with baseline characteristics similar to ours (e.g. comparable age, ECOG‐PS, histological subtype and rates of B‐symptoms and advanced‐stage disease), although they had a higher burden of comorbidities (median CIRS‐G 6, range 0–23). CR and TRM rates were also comparable (74% and 8%, respectively), although most patients (79%) in the NLG cohort received CHOP (cyclophosphamide, doxorubicin, vincristine, prednisone) as potentially curative therapy instead of ABVD.^9^ In the original study, the Frailty Score effectively classified patients as fit, unfit and frail, with a strong impact on both 5‐year OS and PFS. The score nicely predicted 5‐year OS in our cohort, as well, and emerged as an independent predictor of OS in the MVA, beside age and advanced stage according to GHSG classification, highlighting the value of a comprehensive risk stratification beyond chronological age.

At a median follow‐up of just over 5 years (65.5 months), 5‐year OS and PFS were 78.5% and 65%, respectively, higher than in many published series. For instance, Evens et al., reported a 5‐year OS and PFS rates of 58% and 44%, respectively, but their population had more advanced disease (64% cases) and higher comorbidity burden, as previously mentioned.^2^ In contrast, the FIL prospective randomized trial comparing VEPEMB and ABVD showed better outcomes (5‐year OS 77% and PFS 70% for ABVD), but frail patients were excluded.^16^ Most of our patients (87.8%) received AVD‐based therapies, associated with higher CR rates (87.1% vs. 46.7% with other regimens; *p* = 0.001) and significantly improved OS (69.1% vs. 35.3%, *p* = 0.001) and PFS (61.8% vs. 23.5%, *p* = 0.002). However, treatment intensity in our cohort was associated with frequent toxicities: infections and grade 3–4 haematological toxicities occurred in over half of the patients (54% and 62.8%, respectively), despite the extensive use of G‐CSF, with higher incidence in older subgroups. Pneumonia was observed in 20.4% of patients, mostly among those with grade 3–4 haematological toxicity (70%), and significantly impacted OS (*p* = 0.009). BLT occurred in 12 patients (9.7% of those receiving bleomycin), without age differences (*p* = 0.96). Its relatively low incidence may be partly due to the retrospective data collection but may also reflect diagnostic variability, due to the lack of consensus on the definition, as BLT rates in the literature range from 5% to 31%, and increase with age.^2^, ^17^, ^18^, ^19^, ^20^ In our cohort, 46 patients with advanced‐stage disease received more than two cycles of full ABVD without bleomycin omission. This can be partly explained by the fact that most patients (32/46, 69.6%) were treated before 2016, prior to the publication of the RATHL (Risk‐Adapted Therapy in Hodgkin Lymphoma) trial, that demonstrated the safety and efficacy of PET2‐guided bleomycin omission in advanced cHL.^21^ Among the 14 patients treated from 2016 onwards, most were aged 60–68 years (10/14, 71.4%), which may have influenced clinicians' decisions to continue bleomycin; two patients had a positive PET2 and continued ABVD because treatment intensification was not feasible. Notably, no BLT was observed in the 14 patients who would have been candidates for bleomycin discontinuation. However, current evidence consistently supports the omission of bleomycin after two ABVD cycles in all patients aged >60 years, irrespective of stage and PET2 results.^19^

Finally, it is known that combined regimens may improve outcomes in cHL compared to ABVD.^22^, ^23^ In the ECHELON‐1 trial subgroup analysis focused on patients aged ≥60 years with stage III–IV cHL, BV‐AVD was associated with a non‐significant improvement in 5‐year PFS compared to ABVD (67.1% vs. 61.6%; *p* = 0.443), at the cost of increased toxicity, including grade 3–4 neuropathy (18% vs. 3%) and febrile neutropenia (37% vs. 17%).^23^ Sequential BV‐AVD appeared better tolerated in older patients.^22^ More recently, the combination of nivolumab and AVD demonstrated superior efficacy and tolerability compared to BV‐AVD, particularly in older patients (2‐year PFS: 89% vs. 64%, *p* = 0.001; 2‐year OS: 96% vs. 85%, *p* = 0.005).^24^, ^25^ The use of the PET2‐guided BrECADD (brentuximab vedotin, etoposide, cyclophosphamide, doxorubicin, dacarbazine and dexamethasone) regimen in older patients with advanced‐stage cHL has become recently available from the phase II GHSG HD21 trial.^26^ In 83 patients aged 61–75 years, BrECADD proved to be feasible and effective, with CR rates of 82%, 2‐year PFS and OS rates exceeding 90%, and an acceptable safety profile, with no cases of TRM and manageable—although frequent—haematological toxicities (96% grade ≥3 leucopenia, 86% grade ≥3 thrombocytopenia, 55% febrile neutropenia).^26^

These results support the potential future role of nivolumab‐AVD regimen as new standard of care for older patients with advanced‐stage cHL who are eligible for anthracycline‐based therapy, with the intensive BrECADD option in selected cases.^24^, ^25^, ^26^

Our study has several limitations. Its single‐centre, retrospective design may have resulted in incomplete data and limits the generalizability of the findings. In addition, the long patients' inclusion period (>20 years) may have introduced heterogeneity in patients' management and supportive care practices. However, most patients received AVD‐based therapy, which has remained the standard of care for cHL for decades, and G‐CSF was used in most cases, suggesting a relatively consistent approach to the prevention and management of treatment‐related neutropenia and infectious complications over time. Accordingly, temporal changes are unlikely to have substantially affected overall outcomes. The lack of a formal assessment of ADL represents an additional limitation, as functional impairment may further refine pretreatment risk stratification.

Given the clear survival benefits of effective and potentially curative therapies, undertreatment based solely on chronological age risks to compromise the outcomes of older cHL patients. Treatment decisions should instead rely on objective fitness assessments. We validated the Frailty Score as an effective tool for prognostic stratification in older cHL patients receiving standard therapies; however, this score was developed retrospectively and requires prospective validation in larger multicentre cohorts, even in the context of the new first‐line schedules.^9^ The score may help identify ‘frail’ patients with poor life expectancy when treated with conventional therapeutic approaches, for whom alternative strategies should be considered and quality of life prioritized as a primary therapeutic goal. In contrast, for ‘unfit’ patients, dose adjustments should be considered in order to preserve treatment efficacy while maintaining an adequate dose intensity. Therefore, following the example of the FIL experience in elderly DLBCL patients,^5^ a prospective multidimensional geriatric assessment—including evaluation of ADL and comorbidities—is essential to guide the therapeutic choices, tailored to each patient. FIL has recently completed a study in this setting, and its results are eagerly awaited to support the development of a fitness‐adapted treatment algorithm for older patients with cHL. Finally, considering the recent results of checkpoint inhibitor‐based combinations, with or without chemotherapy,^24^, ^25^ the therapeutic armamentarium should soon be enriched with effective alternative treatment options for less fit patients.

## AUTHOR CONTRIBUTIONS

IDG conceived the study and supervised the work; SL and GMA collected data, wrote the manuscript and reviewed the literature; GMA, MP, GA, GMDE, IDG managed the patients; GMA performed the statistical analysis of data and created figures and tables; IDG, MM, AP critically reviewed and commented on the manuscript. All authors took part in writing the manuscript and reviewed and approved the final version.

## FUNDING INFORMATION

No support from any funding agency in the public commercial or not‐for‐profit sector was received for this research.

## CONFLICT OF INTEREST STATEMENT

The authors declare no conflicts of interest.

## ETHICS STATEMENT

Compliance with Italian ethical standards: approval of the Ethics Committee Lazio Area 1 (Rif. 7266, Prot. 0849/2023, date 18/10/2023). The study was conducted in accordance with the Declaration of Helsinki.

## CONSENT TO PARTICIPATE

Given its retrospective design and the use of anonymized data, the requirement for informed consent was waived by the local Ethics Committee.

## Supporting information


**Table S1.** Comorbidities recorded at diagnosis.
**Table S2.** Type of first‐line treatment and modification.
**Table S3.** Clinical and treatment characteristics of patients with therapy‐related mortality or early loss to follow‐up during first‐line therapy.
**Table S4.** Multivariate analysis with patient and disease related factor for PFS.
**Table S5.** Multivariate analysis with patient and disease related factor for OS.

## Data Availability

Data may be made available from the corresponding author upon reasonable request.
