# Supplementary material for: Prognostic impact of treatment‐related and geriatric factors in older patients with classic Hodgkin lymphoma: A real‐life cohort study
Source: Br J Haematol. 2026 Feb 20;208(4):1296–305. doi: 10.1111/bjh.70390 (PMC13071466; doi:10.1111/bjh.70390)
Supplement: Supplementary file 1 — Table S1. Comorbidities recorded at diagnosis. Table S2. Type of first‐line treatment and modification. Table S3. Clinical and treatment characteristics of patients with therapy‐related mortality or early loss to follow‐up during first‐line therapy. Table S4. Multivariate analysis with patient and disease related factor for PFS. Table S5. Multivariate analysis with patient and disease related factor for OS. [file BJH-208-1296-s001.docx]

**Prognostic impact of treatment-related and geriatric factors in older patients with classic Hodgkin lymphoma: a real-life cohort study.**

**Authors:** Silvio Ligia^1†^, Giovanni Manfredi Assanto^1,2^, Tania Soriano^1,2^, Luca Vincenzo Cappelli^1,2^, Mauro Passucci^1^, Giorgia Annechini^2^, Gianna Maria D’Elia^2^, Alessandro Pulsoni^1,3^, Maurizio Martelli^1,2^, Ilaria Del Giudice^1,2^*

**Affiliations:**

^1^Hematology, Department of Translational and Precision Medicine, Sapienza University of Rome, Rome, Italy;

^2^Hematology, AOU Policlinico Umberto I, Rome, Italy;

^3^Hematology Unit, S. Maria Goretti Hospital, Latina, Italy.

**SUPPLEMENTARY MATERIALS**

**Statistical Analysis**

Statistical analysis was performed using IBM SPSS Statistics v.25tm software. Categorical variables were reported as absolute numbers and relative percentages to the total population and individual subdivisions; continuous variables were reported as median value, range (95% CI), and mean. Pearson's chi-square test and Fisher's exact test were used for univariate analysis of categorical variables, while the Wilcoxon test and Mann-Whitney test were used for univariate analysis of continuous variables to assess the distribution of population characteristics and to verify the association of characteristics with mortality and disease progression. Variables were initially assessed through univariate logistic regression analysis to define the impact of each characteristic on the risk of complications, progression, and/or death. Univariate analysis using the Kaplan-Meier model was conducted on significant characteristics in binary logistic regression (p<0.05) in relation to PFS and OS. The significance of Kaplan-Meier curves was calculated using the Log-Rank (Mantel-Cox) test. Subsequently, multivariate analysis was conducted using conditional stepwise Cox regression, including factors resulting significant in univariate analysis.

**SUPPLEMENTARY TABLES**

**Table S1** - Comorbidities recorded at diagnosis.

| Characteristic | | Age categories | | | | | | | | p-value |
| --- | --- | --- | --- | --- | --- | --- | --- | --- | --- | --- |
|  |  | 60-69 Years (N=71) | | 70-79 Years (N=50) | | ≥80 Years (N=19) | | Total  (N=140) | |  |
|  |  | N | % | N | % | N | % | N | % |  |
| Comorbidities | No | 16 | 22.5% | 0 | 0.0% | 0 | 0.0% | 16 | 11.4% | **<0.001** |
|  | Yes | 55 | 77.5% | 50 | 100.0% | 19 | 100.0% | 124 | 88.6% |  |
| Cardiovascular disease | | 36 | 50.7% | 37 | 74.0% | 14 | 73.7% | 87 | 62.1% | **0.018** |
| Previous myocardial infarction | | 4 | 5.6% | 2 | 4.0% | 4 | 21.1% | 10 | 7.1% | **0.038** |
| Pulmonary comorbidities | | 19 | 26.8% | 15 | 30.0% | 6 | 31.6% | 40 | 28.6% | 0.88 |
| Diabetes | | 19 | 26.8% | 15 | 30.0% | 6 | 31.6% | 40 | 28.6% | **0.019** |
| Metabolic syndrome | | 3 | 4.2% | 3 | 6.0% | 2 | 10.5% | 8 | 5.7% | 0.57 |
| Autoimmune diseases | | 7 | 9.9% | 13 | 26.0% | 3 | 15.8% | 23 | 16.4% | 0.06 |
| Previous neoplasia | | 6 | 8.5% | 17 | 34.0% | 4 | 21.1% | 27 | 19.3% | **0.002** |

**Table S2** - Type of first-line treatment and modification.

| Characteristic | | Age Categories | | | | | | | | p-value |
| --- | --- | --- | --- | --- | --- | --- | --- | --- | --- | --- |
|  |  | 60-69 Years (N=71) | | 70-79 Years (N=50) | | ≥80 Years (N=19) | | Total  (N=140) | |  |
|  |  | N | % | N | % | N | % | N | % |  |
| First-line treatment | ABVD | 56 | 78.9% | 36 | 72.0% | 8 | 42.1% | 100 | 71.4% | **<0.001** |
|  | AVD | 1 | 1.4% | 1 | 2.0% | 2 | 10.5% | 4 | 2.9% |  |
|  | ABVD+AVD | 8 | 11.3% | 6 | 12.0% | 2 | 10.5% | 16 | 11.4% |  |
|  | BV+AVD | 1 | 1.4% | 2 | 4.0% | 0 | 0.0% | 3 | 2.1% |  |
|  | VEPEMB | 3 | 4.2% | 5 | 10.0% | 0 | 0.0% | 8 | 5.7% |  |
|  | RT only | 1 | 1.4% | 0 | 0.0% | 2 | 10.5% | 3 | 2.1% |  |
|  | PROVECIP | 1 | 1.4% | 0 | 0.0% | 5 | 26.3% | 6 | 4.3% |  |
| Bleomycin withdrawn | | 10 | 15.6% | 9 | 21.4% | 2 | 20.0% | 21 | 18.1% | 0.74 |
| Radiotherapy | | 39 | 54.9% | 27 | 54.0% | 6 | 31.6% | 72 | 51.4% | 0.18 |
| Treatment cycle delay | | 28 | 40.0% | 21 | 42.0% | 11 | 61.1% | 60 | 43.5% | 0.26 |
| Dose reduction | | 8 | 11.4% | 6 | 12.2% | 6 | 33.3% | 20 | 14.6% | **0.05** |
| Early treatment discontinuation | | 6 | 8.7% | 8 | 16.3% | 8 | 44.4% | 22 | 16.2% | **0.001** |

Abbreviations: ABVD, doxorubicin bleomycin vinblastine and dacarbazine; BV, brentuximab vedotin; VEPEMB, vinblastine cyclophosphamide procarbazine prednisone etoposide mitoxantrone and bleomycin; RT, radiotherapy; PROVECIP, procarbazine vinblastine cyclophosphamide and prednisone.

**Table S3 –** Clinical and treatment characteristics of patients with therapy-related mortality or early loss to follow-up during first-line therapy

| Pt. | Age (years), Sex | CIRS-G  score | Main comorbidities | HL stage | Therapy regimen (cycles) | Response | Treatment complications | Final cause of death / Lost to follow up |
| --- | --- | --- | --- | --- | --- | --- | --- | --- |
| 1. | 67, M | 2 | - | IV B,E | ABVD (x2) | NA | - | Ischemic stroke after the 2^nd^ cycle |
| 2. | 72, F | 6 | HTA | IV B,E | BV+AVD (x3) | Negative PET2 | CHF, G3-4 neutropenia, neuropathy | Septic shock after the 3^rd^ cycle |
| 3. | 80, F | 9 | AMI, HTA, smoke | III B | ABVD (x2) + AVD (x1) | Negative PET2 | Infection, G3-4 neutropenia | Pneumonia after the 3^rd^ cycle |
| 4. | 73, M | 14 | DM, HTA, autoimmune | III A | ABVD (x2) + AVD (x2) | Negative PET2 | CHF, G3-4 neutropenia, pneumonia | Death ~3 weeks after therapy interruption (multiple toxicities) |
| 5. | 86, F | 12 | HTA, COPD, DM, smoke | III B | PROVECIP (x4) | NA | NR | Death ~4 weeks after the EOT, cause NR |
| 6. | 73, M | 6 | HTA, smoke | III A | ABVD (x5) | Negative PET2 | Infection, G3-4 neutropenia | Pneumonia after the 5^th^ cycle |
| 7. | 65, F | 0 | - | III B | ABVD (x2) | NA | Infection | Sepsis, ~2 weeks after the end of 2^nd^ cycle |
| 8. | 84, M | 9 | Previous cancer, DM, AMI, smoke | IV B,E | PROVECIP (x2) | NA | G3-4 neutropenia | Lost to follow up during therapy |
| 9. | 81, M | 3 | AMI | II A | ABVD (x2) + AVD (x2) + RT | Negative PET2 | Infection, G3-4 neutropenia | Lost to follow up after EOT |
| 10. | 74, F | 4 | HTA | IV A,E | ABVD (x4) | Negative PET2 | Infection, G3-4 neutropenia | Lost to follow up during therapy |
| 11. | 76, M | 5 | Previous cancer, HTA | IV A,E | ABVD (x4) | Negative PET2 | Pneumonia (resolved), G3-4 neutropenia | Lost to follow up during therapy |

Abbreviations: NA, not assessed; NR, not reported; CIRS-G, Cumulative Illness Rating Scale-Geriatric; M, male; F, female; A, absence of B symptoms; B, presence of B symptoms; E, extranodal disease; ABVD, doxorubicin bleomycin vinblastine and dacarbazine; BV, brentuximab vedotin; RT, radiotherapy; PROVECIP, procarbazine vinblastine cyclophosphamide and prednisone; PET2, interim PET after 2 therapeutic cycles; EOT, end of treatment; G, grade; HTA, arterial hypertension; DM, diabetes mellitus; CHF, congestive heart failure; AMI, acute myocardial infarction; COPD, chronic obstructive pulmonary disease.

**Table S4** - Multivariate analysis with patient and disease related factor for PFS

| Characteristics included in Cox Regression model for PFS | | | | | | | | | |
| --- | --- | --- | --- | --- | --- | --- | --- | --- | --- |
|  | | B | SE | Wald | gl | Sign. | HR | 95.0% CI per HR | |
|  |  |  |  |  |  |  |  | Inferior | Superior |
| Step 1 | Age ≥80 years | .905 | .437 | 4.294 | 1 | .038 | 2.472 | 1.050 | 5.818 |
|  | ECOG-PS 2-4 | -.023 | .417 | .003 | 1 | .956 | .977 | .432 | 2.213 |
|  | Age ≥70 years | -.826 | .772 | 1.145 | 1 | .285 | .438 | .096 | 1.988 |
|  | Frailty Score | .490 | .397 | 1.523 | 1 | .217 | 1.633 | .749 | 3.557 |
|  | Advanced Stage (III-IV) | -.568 | .662 | .735 | 1 | .391 | .567 | .155 | 2.075 |
|  | B-Symptoms | .092 | .377 | .060 | 1 | .806 | 1.097 | .524 | 2.296 |
|  | Splenomegaly | .141 | .337 | .175 | 1 | .675 | 1.152 | .595 | 2.230 |
|  | Extranodal Disease | .898 | .452 | 3.950 | 1 | .047 | 2.454 | 1.013 | 5.950 |
|  | GHSG Risk | .705 | .415 | 2.879 | 1 | .090 | 2.023 | .896 | 4.564 |
| Step 7 | Age ≥80 Years | .663 | .171 | 15.113 | 1 | **.000** | 1.941 | 1.390 | 2.713 |
|  | Extranodal Disease | .920 | .416 | 4.881 | 1 | **.027** | 2.508 | 1.109 | 5.670 |
|  | GHSG Risk (High) | .513 | .182 | 7.941 | 1 | **.005** | 1.669 | 1.169 | 2.384 |

Abbreviations: ECOG-PS, Eastern Cooperative Oncology Group performance status; GHSG, German Hodgkin Study Group.

**Table S5 -** Multivariate analysis with patient and disease related factor for OS

| Characteristics included in Cox Regression model for OS | | | | | | | | | |
| --- | --- | --- | --- | --- | --- | --- | --- | --- | --- |
|  | | B | SE | Wald | gl | Sign. | HR | 95.0% CI per HR | |
|  |  |  |  |  |  |  |  | Inferior | Superior |
| Step 1 | Age ≥80 years | 1.531 | .483 | 10.049 | 1 | .002 | 4.621 | 1.794 | 11.904 |
|  | Cardiovascular comorbidity | .379 | .357 | 1.127 | 1 | .288 | 1.460 | .726 | 2.938 |
|  | Age ≥70 years | -2.078 | 1.000 | 4.320 | 1 | .038 | .125 | .018 | .888 |
|  | CIRS-G >8 | -.158 | .659 | .058 | 1 | .810 | .854 | .235 | 3.106 |
|  | ECOG-PS 2-4 | -.587 | .545 | 1.161 | 1 | .281 | .556 | .191 | 1.617 |
|  | Frailty Score (High) | 1.193 | .733 | 2.647 | 1 | .104 | 3.295 | .784 | 13.860 |
|  | Advanced Stage (III-IV) | .862 | .961 | .806 | 1 | .369 | 2.369 | .360 | 15.572 |
|  | B-symptoms | -.439 | .427 | 1.057 | 1 | .304 | .645 | .280 | 1.488 |
|  | EORTC Risk | -.592 | .795 | .555 | 1 | .456 | .553 | .116 | 2.628 |
|  | GHSG Risk | .580 | .527 | 1.209 | 1 | .271 | 1.785 | .636 | 5.015 |
|  | ACA index | .071 | .196 | .132 | 1 | .717 | 1.074 | .731 | 1.577 |
| Step 8 | Age ≥80 years | 1.198 | .388 | 9.533 | 1 | **.002** | 3.315 | 1.549 | 7.094 |
|  | Age ≥70 years | -1.446 | .686 | 4.445 | 1 | **.035** | .235 | .061 | .903 |
|  | Frailty score (High) | .795 | .324 | 6.028 | 1 | **.014** | 2.214 | 1.174 | 4.174 |
|  | GHSG Risk (High) | .502 | .210 | 5.687 | 1 | **.017** | 1.651 | 1.093 | 2.494 |

Abbreviations: CIRS-G, Cumulative Illness Rating Scale-Geriatric; GHSG, German Hodgkin Study Group; ECOG-PS, Eastern Cooperative Oncology Group performance status; EORTC, European Organisation For Research And Treatment Of Cancer.
